# The Role of Gut and Airway Microbiota in Pulmonary Arterial Hypertension

**DOI:** 10.3389/fmicb.2022.929752

**Published:** 2022-07-13

**Authors:** Linlin Huang, Hongdie Zhang, Yijun Liu, Yang Long

**Affiliations:** ^1^Experimental Medicine Center, The Affiliated Hospital of Southwest Medical University, Luzhou, China; ^2^Department of Endocrinology and Metabolism, The Affiliated Hospital of Southwest Medical University, Luzhou, China; ^3^Metabolic Vascular Disease Key Laboratory of Sichuan Province, Luzhou, China; ^4^Sichuan Clinical Research Center for Nephropathy, Luzhou, China; ^5^Academician (Expert) Workstation of Sichuan Province, The Affiliated Hospital of Southwest Medical University, Luzhou, China

**Keywords:** pulmonary arterial hypertension, gut microbiota, airway microbiota, gut-lung axis, microbiota-based therapy

## Abstract

Pulmonary arterial hypertension (PAH) is a severe clinical condition that is characterized pathologically by perivascular inflammation and pulmonary vascular remodeling that ultimately leads to right heart failure. However, current treatments focus on controlling vasoconstriction and have little effect on pulmonary vascular remodeling. Better therapies of PAH require a better understanding of its pathogenesis. With advances in sequencing technology, researchers have begun to focus on the role of the human microbiota in disease. Recent studies have shown that the gut and airway microbiota and their metabolites play an important role in the pathogenesis of PAH. In this review, we summarize the current literature on the relationship between the gut and airway microbiota and PAH. We further discuss the key crosstalk between the gut microbiota and the lung associated with PAH, and the potential link between the gut and airway microbiota in the pathogenesis of PAH. In addition, we discuss the potential of using the microbiota as a new target for PAH therapy.

## Introduction

Pulmonary arterial hypertension (PAH) is a group of diseases characterized by a progressive increase in pulmonary artery pressure, which can lead to irreversible right heart failure. The pulmonary artery pressure of 25 mmHg or higher was defined as pulmonary arterial hypertension (Poch and Mandel, 2021). Pulmonary arterial hypertension is further classified as idiopathic, hereditary, drug-induced, and disease-related PAH (Sockrider, 2021). Heterozygous mutations in bone morphogenetic protein receptor 2 (BMPR2) have been reported in 53–86% of hereditary pulmonary arterial hypertension cases and in 14–35% of idiopathic PAH patients (Gräf et al., 2018). In addition, disease-related PAH such as congenital heart disease, connective tissue disease, HIV, and schistosomiasis can cause destruction or narrowing of the pulmonary arteries, resulting in elevated pulmonary artery pressure (Ruopp and Cockrill, 2022). The prevalence of PAH has been reported to be 15–50 per million in the United States and Europe (Beshay et al., 2020). A systematic review from multiple national publications reports that in adults, the incidence of PAH is ≈5.8 per million and the prevalence of PAH is ≈47.6–54.7 per million (Leber et al., 2021). At present, most of the epidemiological data on PAH are based on studies of its etiology and vary between different regions of the world. PAH has a low incidence but serious consequences. Most patients with PAH remain incurable. Therefore, it is important to find new potential treatments for PAH.

In recent years, investigators increasingly focused on the microbial–host interactions. The microbiota consists of complex communities of bacteria, archaea, fungi, viruses, and protists that colonize multiple parts of the body, such as the gastrointestinal tract, oral, skin, and genitourinary tract (Stappenbeck and Virgin, 2016). Dysbiosis, defined as an imbalance in the composition of the microbiota, has been associated with several diseases such as chronic obstructive pulmonary disease, hypertension, heart failure, obesity, and inflammatory bowel disease et al. (Halfvarson et al., 2017; Li et al., 2017; Callejo et al., 2018; Cui et al., 2018; Bowerman et al., 2020; Kim M. H. et al., 2020). Also, dysbiosis in gut and airway microbiota has been observed in patients with PAH (Kim S. et al., 2020). Both the intestine and the lungs are major organs in direct communication with the outside world; they have the same embryonic origin and structural similarities. Therefore, it is not surprising that these two locations may have interactions in the development of health and disease, but the exact mechanisms are not known. In fact, it has been shown that there is crosstalk between the gut microbiota and the lungs, and the connection has been named the gut–lung axis (He et al., 2017). Lipopolysaccharide (LPS) and short-chain fatty acids (SCFAs) produced by intestinal microorganisms have been reported to play an important role in regulating immune tone in lungs (Liu et al., 2021). On the other hand, it has been demonstrated that lung infections in mice also cause gut dysbiosis (Groves et al., 2018). Recent studies have found that changes in the structure and function of gut and airway microbiota are associated with PAH (Kim S. et al., 2020; Zhang et al., 2020). In this review, we will provide an overview of the gut and airway microbial alterations reported in PAH. Then, we will discuss the mechanistic evidence explaining the connections between dysbiosis in gut and airway microbiota and PAH. In addition, we will point out the possibility of targeting the microbiome and its related components as a new therapeutic option for PAH.

## Inflammation in PAH

The characteristic pathological feature of PAH is pulmonary vascular remodeling involving all layers of the vessel wall, such as intima (endothelial cells), media (pulmonary artery smooth muscle cells-PASMCs), and adventitia (fibroblasts) (Thenappan et al., 2018). Hyperproliferation of vascular cells and deposition of the extracellular matrix leads to thickening of the arterial wall and muscularization of small non-muscular arteries and reducing the compliance of the pulmonary vasculature (Thenappan et al., 2018). There is growing evidence that pulmonary perivascular inflammation plays an important role in the early stages of PAH or pulmonary vascular remodeling. Significantly increased infiltration of macrophages, mast cells, T lymphocytes, B lymphocytes, dendritic cells, and monocytes has been found in the pulmonary vasculature of patients with PAH, and have a vital effect on pulmonary perivascular inflammation and pathological processes of PAH (Savai et al., 2012; Zhu et al., 2021; Wang et al., 2022). Increased macrophages, mast cells, and B cells could accelerate inflammatory responses and pulmonary vascular remodeling in PAH (Ni et al., 2022). Accordingly, the expression levels of various cytokines and chemokines have been observed to be elevated in PAH patients and animal models. Increased expression of interleukin (IL)-1β, IL-6, IL-1 receptor (R)1, and IL6R and their role in pulmonary vascular remodeling have been reported in patients with idiopathic PAH and hypoxia-induced PAH mice (Parpaleix et al., 2016; Tamura et al., 2018). In hypoxia-induced PAH mice, IL-33, a member of the IL-1 family of cytokines, binds to the membrane receptors ST2, and initiates pulmonary vascular remodeling by upregulating the expression of hypoxia-inducible factor-1α (HIF-1α) and vascular endothelial growth factor (VEGF) (Liu et al., 2018). Abid et al. found that chemokine receptor (CCR) 2 and CCR5 expression were increased in PASMCs and perivascular macrophages of PAH patients and promoted PASMCs growth (Abid et al., 2019). The expression of high mobility group box-1 (HMGB1) and toll-like receptor 4 (TLR4) was significantly increased in the lungs of PAH patients, and HMGB1 promoted PAH through activation of TLR4 (Ranchoux et al., 2017; Goldenberg et al., 2019). In addition, endothelial dysfunction is a major pathophysiological mechanism of PAH, resulting from the altered production of endothelial vasoactive mediators. The expression of intercellular cell adhesion molecule-1, vascular cell adhesion molecule-1 (VCAM-1), and E-selectin is significantly upregulated in the pulmonary arteries of idiopathic PAH patients (Le Hiress et al., 2015). In a word, these data strongly suggest that perivascular inflammation and pulmonary vascular remodeling play an important role in the development of PAH. However, the specific mechanisms that cause perivascular inflammation and pulmonary vascular remodeling are still being explored, and recent studies have begun to focus on the role of microorganisms and their metabolites in PAH.

## Gut–Lung Axis in PAH

The majority of the microbiota is colonized in the intestinal tract, which is usually dominated by five bacterial phyla, such as *Bacteroidetes, Firmicutes, Proteobacteria, Actinobacteria*, and *Tenericutes* (Stappenbeck and Virgin, 2016; Almeida et al., 2019). Of these, *Firmicutes* and *Bacteroidetes* are the two most common phyla and constitute more than 90% of the gut microbiota. It has been reported that the *Firmicutes/Bacteroidetes* ratio, a classic biomarker of dysbiosis, is increased in individuals with obesity, diabetes, and cardiovascular disease (Mariat et al., 2009; Koliada et al., 2017; Tsai et al., 2021). The proportion of intestinal *F/B* was higher in most of the sugen/hypoxia, monocrotaline (MCT), or hypoxia-induced PAH animal models (Callejo et al., 2018; Sanada et al., 2020; Sharma et al., 2020b; Hong et al., 2021; Luo et al., 2021). Kim et al. compared the fecal microbiome of PAH patients and reference subjects by shotgun metagenomics and reported distinct gut microbiome composition in the PAH patients with reduced alpha diversity, richness, and evenness (Kim S. et al., 2020). In contrast, Sharma et al. reported that the *F/B* ratio was reduced in the pure hypoxia-induced PAH mice (Sharma et al., 2020a). However, in a pilot study of the gut microbiome in 20 PAH patients and 20 paired control subjects, no differences in microbial abundance and diversity are reported in PAH patients with no significant differences in alpha diversity, beta diversity, and *F/B* ratio (Jose et al., 2022). Several factors may contribute to these inconsistent findings. First, the pilot study is a pair-controlled prospective cohort study, and a multitude of potentially confounding factors such as obesity, dietary intake, environmental factors, and circadian rhythm, was rigorously controlled by using paired enrollment scheme. Moreover, the sample sizes were both limited in these two studies of human gut microbiota and, consequently, these studies were underpowered to detect a difference in gut microbiota diversity in PAH patients.

Decreased SCFAs-producing bacteria may be one of the most important clues for the roles of gut microbiota dysbiosis in the PAH process. Lower rates of butyrate-producing bacteria (*Coprococcus, Butyrivibrio, Lachnospiraceae*, and *Eubacterium*) and propionate-producing bacteria (*Akkermansia* and *Bacteroides*) in PAH patients (Kim S. et al., 2020; Jose et al., 2022) (Table 1). Similarly, the abundance of *Akkermansia* and *Bacteroides* was reduced in sugen/hypoxia rats compared to the control group (Sanada et al., 2020). SCFAs have been demonstrated to have vital effects on the host immune response and inflammation regulation (Kemter and Nagler, 2019). SCFAs can activate G protein-coupled receptors (GPCRs, such as GPR43, GPR41, and GPR109a) or act as histone deacetylase (HDAC) inhibitors to promote the production of regulatory T cells (Tregs) through epigenetic modifications (Arpaia et al., 2013; Smith et al., 2013; Trompette et al., 2014); and Tregs can protect the vascular endothelium against PAH (Tamosiuniene et al., 2011). Liu et al. found that GPR43 and GPR41 are expressed in human alveolar primary cells and regulated by intestinal-derived LPS, and directly verified the transmission of LPS and SCFA from the intestine to the lung using germ-free mice (Liu et al., 2021). It is reported that butyrate treatment can reverse the increase of CD68+ and CD163+ macrophages in the lungs of PAH rats induced by hypoxia (Karoor et al., 2021). Butyrate also exerts its anti-inflammatory effects by inhibiting the development of T helper 17 (Th17) cells and down-regulating VCAM-1 and CX3C chemokine Fractalkine in endothelial cells (Li et al., 2018; Chen et al., 2019; Karoor et al., 2021). In addition to immune regulation, Karoor and Kim et al. found that butyrate inhibited hypoxia-induced angiogenesis by decreasing the expression of VEGF-α and HIF-1α (Kim et al., 2007; Karoor et al., 2021). Furthermore, butyrate plays a significant role in maintaining the integrity of pulmonary microvascular endothelial and gastrointestinal barrier (Plöger et al., 2012; Karoor et al., 2021). Conversely, enrichment of the *Collinsella* in the intestine of PAH patients increases intestinal permeability by down-regulating tight junction proteins and promotes the production of epithelial IL-17A (Kim et al., 2018; Kim S. et al., 2020). Serotonin (5-HT), a key mediator of PAH, has been associated with the PAH phenotype of BMPR2 deficient mice by inhibiting BMP signaling, enhanced pulmonary arteries contractile and promoted the PASMCs proliferation (Long et al., 2006; Hood et al., 2017). SCFAs have been found to promote tryptophan hydroxylase 1 transcription and increased 5-HT production in human enterochromaffin cells (Reigstad et al., 2015). Moreover, decreased the proportion of *Lactobacillus* in PAH patients and animal models can also promote 5-HT synthesis (Sharma et al., 2020a).

**Table 1:** Alterations of Microbiota in PAH.

**Reference**	**Origin**	**Sample**	**Enriched microbiota**	**Depleted microbiota**
Kim S. et al. (2020)	Human	Fecal samples	*Firmicutes (Clostridium, Staphylococcus, Blautia, Streptococcus, Roseburia, Rumonococcus), Bacteroidetes (Prevotella), Actinobacteria (Rothia, Bifidobacterium, Collinsella, Coriobacteriales), Proteobacteria (Citrobacter, Desulfovibrio, Enterobacter, Escherichia, Klebsiella, Pseudomonas)*	*Firmicutes (Coprococcus, Butyrivibrio, Clostridia, Lachnospiraceae, Eubacterium, Akkermansia, Lactococcus, Subdoligranulum), Bacteroidetes (Bacteroides), Proteobacteria (Parasutterella)*
Callejo et al. (2018)	Rat	Fecal samples	*Firmicutes (Peptostreptococcaceae)*	*Firmicutes (Butyrivibrio, Aerococcaceae, Syntrophomonadacea), Bacteroidetes (Odoribacter, Butyricimonas, Porphyromonas, Parabacteroides), Proteobacteria (Pasteurellaceae)*
Sanada et al. (2020)	Rat	Fecal samples	*Firmicutes (Coprococcus, Lachnospiraceae, Eubacterium, Allobaculum, Coprostanoligenes, Acetitomaculum, Ruminococcaceae, Faecalibaculum), Bacteroidetes (Prevotellaceae, Parabacteroides), Actinobacteria (Rothia, Bifidobacterium, Parvibacter), Proteobacteria (Parasutterella), Cyanobacteria*	*Firmicutes (Dehalobacterium, Akkermansia, Marvinbryantia, Enterococcus), Bacteroidetes (Bacteroides)*
Hong et al. (2021)	Rat	Fecal samples	*Firmicutes (Clostridia, Allobaculum, Turicibacter, Clostridium), Actinobacteria (Bifidobacterium), Proteobacteria (Ralstonia, Gammaproteobacteria), Candidatus_Saccharimonas*	*Firmicutes (Romboutsia, Lactobacillus, Bacilli), Bacteroidetes (Bacteroidota), Spirochaetota*
Sharma et al. (2020b)	Rat	Fecal samples	*Firmicutes (Clostridiales, Enterococcaceae, Clostridium, Aerococcaceae, Erysipelotrichaceae, Roseburia, Oscillospira), Actinobacteria (Corynebcteriaceae, Corynebcterium), Tenericutes (Mollicutes)*	*Firmicutes (Streptococcaceae, Blautia), Bacteroidetes (S24_7, Bacteroidia), Actinobacteria (Bifidobacterium), Proteobacteria (Enterobacteriales, Enterobacteriaceae, Proteus)*
Luo et al. (2021)	Mice	Fecal samples	*Firmicutes (Lactobacillus, Lactobacillaceae, Christensenella, Erysipelotrichaceae), Bacteroidetes (Marinifilaceae, Rikenellaceae), Actinobacteria (Gordonibacter, Coriobacteriales, Eggerthellaceae), Proteobacteria (Oceanospirillales, Halomonas, Alphaproteobacteria, Rhodospirillales), Melainabacteria*	*Firmicutes (Lachnospiraceae), Bacteroidetes (Bacteroidaceae, Prevotellaceae, Tannerellaceae), Proteobacteria*
Sharma et al. (2020a)	Mice	Fecal samples	*Firmicutes (Oscillospira, Ruminococcus), Bacteroidetes (Prevotella), Proteobacteria*	*Firmicutes (Lactobacillus)*
Zhang et al. (2020)	Human	Pharyngeal swab samples	*Firmicutes (Streptococcaceae, Streptococcus), Proteobacteria (Lautropia, Ralstonia), Fusobacteria (Leptotrichiaceae, Leptotrichia), Chloroflexi*	*Firmicutes (Carnobacteriaceae, Granulicatella), Bacteroidetes (Prevotella, Porphyromonadaceae, Flavobacteriaceae, Alloprevotella, Capnocytophage), Actinobacteria (Rothia), Proteobacteria (Haemophilus), Saccharibacteria, SR1_Absconditabacteria*
Jose et al. (2022)	Human	Fecal samples	*Firmicutes (Anaerostipes rhamnosivorans)*	*Firmicutes (Lachnospiraceae bacteriumGAM79, Amedibacterium intestinale, Ruminococcus bicirculans, Ruminococcus albus)*

Trimethylamine (TMA) is initially generated by gut bacteria as a by-product of the metabolism of dietary nutrients such as choline, and TMA is oxidized to produce trimethylamine N-oxide (TMAO) (Hayase and Jenq, 2020). TMAO has been reported to be associated with the development of atherosclerosis (Hayase and Jenq, 2020). In a meta-analysis of 923 patients at high/very high risk of cardiovascular disease, high levels of TMAO were found to be a possible risk factor for cardiovascular disease (Guasti et al., 2021). A higher proportion of TMA/TMAO-producing intestinal bacteria has been observed in PAH patients and animal models, such as *Clostridium, Desulfovibrio, Enterobacter, Escherichia, Klebsiella, Pseudomonas, Rothia, Prevotella, Clostridium, Staphylococcus, Streptococcus, Anaerostipes rhamnosivorans*, and *Collinsella* (Kim S. et al., 2020; Sharma et al., 2020b; Jose et al., 2022) (Table 1). The expression levels of TMAO and tumor necrosis factor-α and IL-1β in plasma of mice fed the Western diet (a risk factor for PAH) were significantly elevated, while the expression of the anti-inflammatory cytokine IL-10 was decreased, and this change was reversed by the inhibitor of trimethylamine formation (Chen K. et al., 2017; Brittain et al., 2019). Lawrie et al. further found that mice fed with Paigen diet (high fat and high cholesterol) developed PAH in a manner dependent on IL-1 β, suggesting that TMAO may promote the development of PAH through the pro-inflammatory effect of cytokines such as IL-1 β (Lawrie et al., 2011). Recently, TMAO has been found to cause endothelial dysfunction through activation of the hypoxia-induced protein kinase C (PKC)/nuclear factor-kappa B (NF-κB)/VCAM-1 pathway as well as the HMGB1/TLR4 axis (Ma et al., 2017; Singh et al., 2019). Chen et al. reported that TMAO also activated nod-like receptor family pyrin domain containing 3 (NLRP3) inflammasome through sirtuin 3 (SIRT3)/superoxide dismutase 2 (SOD2)/mitochondrial ROS pathway to induce inflammation in endothelial cells and blood vessels (Chen M. L. et al., 2017). At present, the mechanism of TMAO-inducing endothelial dysfunction and vascular inflammation has been widely studied in atherosclerosis, but their specific mechanisms in PAH remain to be elucidated. Additionally, a high proportion of TMAO-producing intestinal microorganisms can also increase the risk of thrombosis, which plays a key role in the development of certain types of PAH (Zhu et al., 2016). However, TMAO is present in seafood, and a recent study found that TMAO not only had no effect on healthy rats but also reduced mortality in rats with heart failure, contradicting the negative effects of TMAO on cardiovascular disease (Gawrys-Kopczynska et al., 2020). Therefore, further investigation should be needed to clarify the impact of TMA/TMAO produced by gut microbes on PAH.

Elevated uric acid levels and reduced arginine bioavailability are associated with the development and prognosis of PAH (Sztormowska-Achranowicz et al., 2020; Savale et al., 2021). It has been reported that treatment with xanthine oxidase inhibitor benzbromarone and dietary supplementation with L-arginine have protective effects against PAH (Sztormowska-Achranowicz et al., 2020). It is commonly assumed that uric acid and arginine originate from diet and endogenous synthesis; however, Kim et al. found that they may also originate from gut microbes. Xanthine oxidase and purine nucleotidase, key enzymes in uric acid synthesis, and purine metabolism, have been found significantly increased in the gut microbiota of patients with PAH (Kim S. et al., 2020). Consistently, high serum uric acid levels are associated with poor prognosis of PAH, and uric acid has been proved to promote the proliferation of PASMCs *in vitro* (Savale et al., 2021). Moreover, dysregulated metabolism of several amino acids, such as arginine, proline, and ornithine, has been found in the gut microbiota of MCT-induced PAH rats and PAH patients (Kim S. et al., 2020; Hong et al., 2021). Consistently, key bacteria which contribute to increased arginine, ornithine, and proline biosynthesis, such as *Blautia, Bifidobacteria*, and *Collinsella* were enriched in PAH (Kim S. et al., 2020; Hong et al., 2021) (Table 1). Furthermore, elevated ornithine transcarbamylase in the gut microbes of PAH patients converts L-ornithine to L-citrulline, may lead to reduced bioavailability of arginine (Kim S. et al., 2020; Sztormowska-Achranowicz et al., 2020).

**Figure 1:**
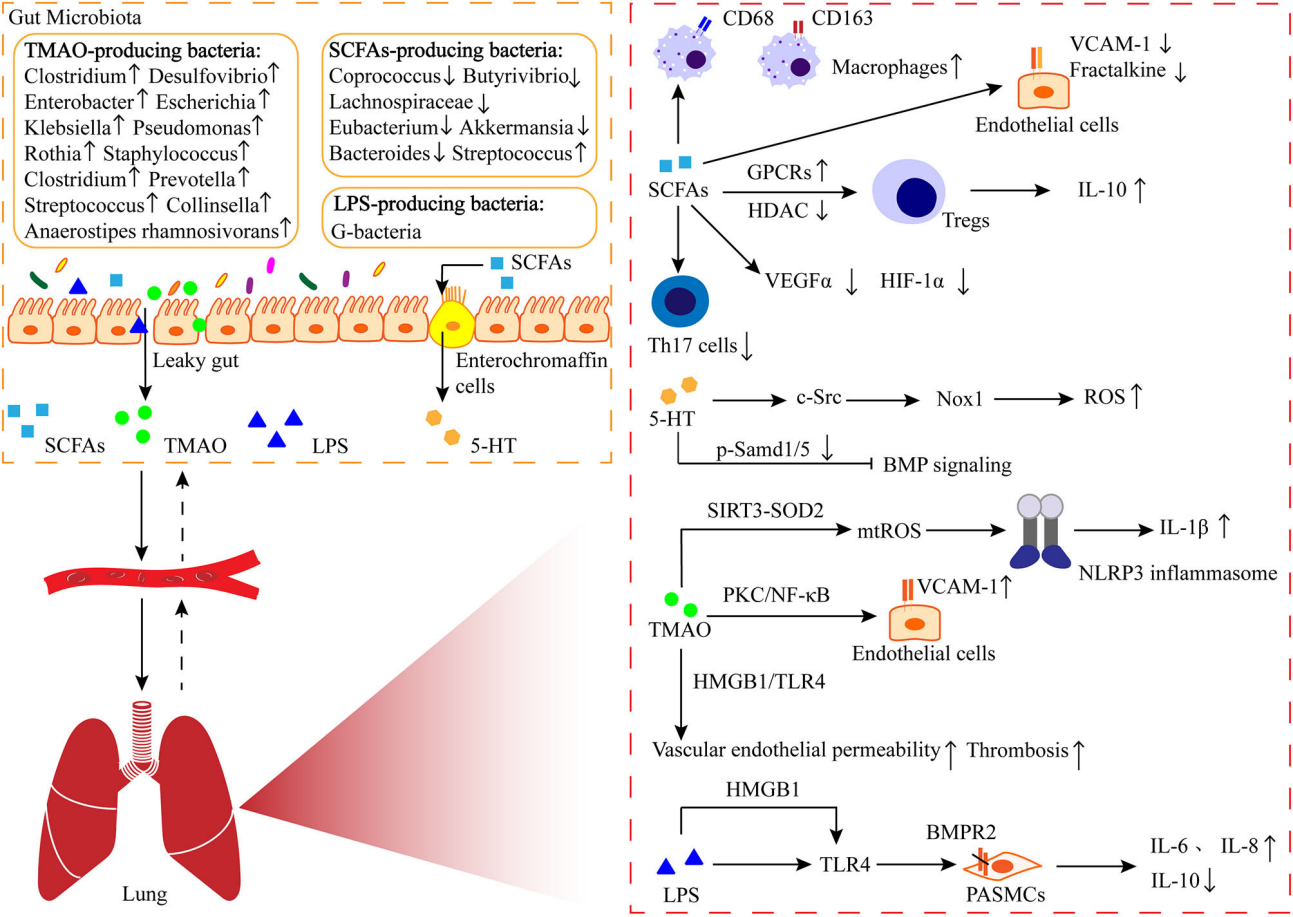
The gut-lung axis in PAH. The metabolites like short-chain fatty acids (SCFAs) produced by intestinal bacteria enter the lungs through the circulation, where they are involved in immune regulation and play a role in perivascular inflammation and pulmonary vascular remodeling. 5-HT: serotonin; TMAO: trimethylamine N-oxide; LPS: lipo-polysaccharide; GPCRs: G protein-coupled receptor; HDAC: histone deacetylase; VCAM-1: vascular cell adhesion molecule-1; VEGFα: vascular endothelial growth factor α; HIF-1α: hypoxia-inducible factor-1α; Nox-1: NADPH oxidase-1; ROS: reactive oxygen species; SIRT3: sirtuin 3; SOD2: superoxide dismutases; NLRP3: nod-like receptor family pyrin domain containing 3; PKC: protein kinase C; NF-κB: nuclear factor-kappa B; HMGB1: high-mobility group box-1; TLR4: toll-like receptor 4; BMPR2: bone morphogenetic protein receptor 2.

In addition, dysbiosis of gut microbiota can increase intestinal permeability, which may lead to translocation of bacteria and bacterial metabolites, as well as an increase in bacterial endotoxins in the plasma (Ranchoux et al., 2017). Endotoxin is a component of the cell wall of “G-bacteria,” also known as LPS, which is the main ligand of TLRs. CD14 has been shown to regulate LPS-induced TLR4 endocytosis, and CD14 is present on the surface of many TLR4-expressing cells (Zanoni et al., 2011). Ranchoux et al. found that serum soluble CD14 was obviously higher in patients with idiopathic PAH and hereditary PAH carrying the BMPR2 mutation compared to control groups (Ranchoux et al., 2017). Acute exposure of Bmpr2+/– mice to LPS induces PAH by promoting ROS-dependent production of inflammatory cytokines (IL-6 and IL-8) compared to WT mice (Soon et al., 2015). LPS has also been reported to bind to HMGB1 and activate TLR4, promoting inflammation and pulmonary vascular remodeling (Ranchoux et al., 2017). Feng et al. found that HMGB1 promotes proliferation and migration of PASMCs by activating extracellular signal-regulated kinase 1/2 (ERK1/2)/dynamin-related protein 1 (Drp1)/Autophagy to inhibit the BMPR2 signaling pathway (Feng et al., 2021). In addition, the previously mentioned TMAO activates HMGB1/TLR4, leading to increased endothelial permeability. This suggests that HMGB1 plays an important role in the influence of microorganisms on PAH and maybe a new target for the treatment of PAH. In addition, Shrestha et al. found that LPS synergistically induced PAH associated with bronchopulmonary dysplasia in experimental mice exposed to hyperoxia (Shrestha et al., 2020) (Figure 1).

## The Microbiota Residing in the Airways Have an Important Impact on PAH

The human respiratory system consists of the upper respiratory tract (such as the nose, pharynx, and larynx) and the lower respiratory tract (such as the trachea and the bronchi at all levels of the lungs). A decade ago, the lower airway was considered a sterile environment, probably because invasive bronchoscopy was not usually performed in healthy lungs and bronchoscopy was suspected of carrying microbial contamination from the oropharynx or nasal cavity. Nevertheless, Hilty et al. used 16S rRNA sequencing to reveal the presence of microbiota in the lower airways, the composition of which differed between chronic obstructive pulmonary disease and asthma patients and reference cohorts (Hilty et al., 2010). Recent studies have also confirmed such results, elucidating the specific role of airway microbiota in respiratory diseases (Budden et al., 2019). The microbiota of healthy lungs appears to be derived from the migration of oral microbiota (Bassis et al., 2015). Moreover, the lungs and intestines have similar epithelial barrier functions and different microbiota. The dominant phyla of the upper respiratory tract, such as *Firmicutes, Bacteroidetes, Proteobacteria, Actinobacteria, Fusobacteria*, and *Saccharibacteria*, partially overlap with the intestinal microbiota (Zhang et al., 2020). Zhang et al. compared the composition of the airway microbiota of PAH patients and reference subjects and found that the airway microbiota of PAH patients had reduced community diversity and increased *F/B* ratio (Zhang et al., 2020). The aforementioned studies suggest that airway microbial dysbiosis plays an important role in the pathogenesis of PAH and that there are close interactions between airway microbiota and the pathological and pathophysiological process of PAH, but a causal relationship cannot yet be established.

**Figure 2:**
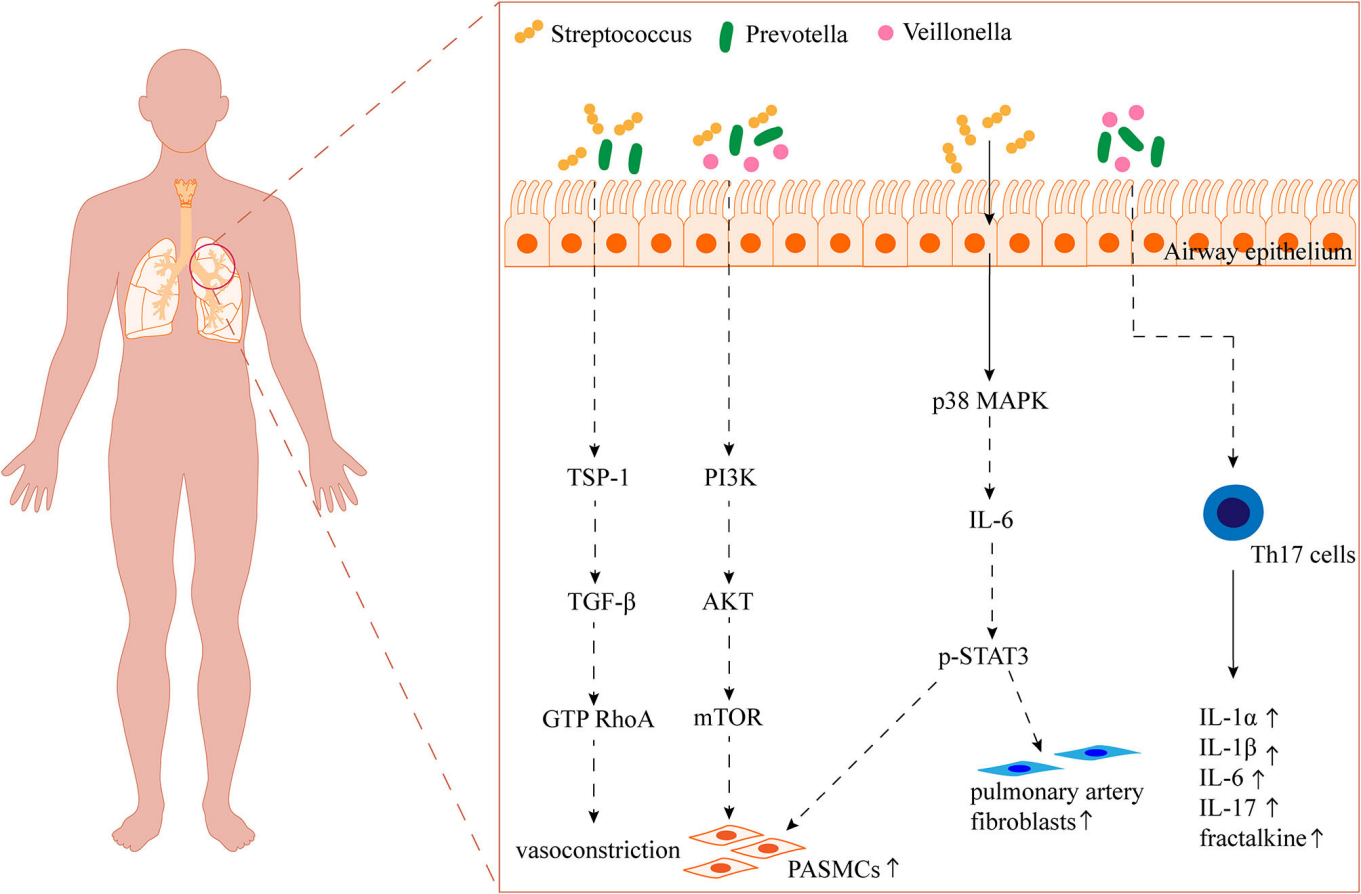
Overview of the potential impact of the airway microbiota on PAH. TSP-1: thrombospondin 1; TGF-β: transforming growth factor-β; RhoA: the small GTP-binding protein; PI3K: phosphatidylinositol 3-kinase; AKT: protein kinase B; mTOR: mammalian target of rapamycin; MAPK: mitogen-activated protein kinase; STAT3: transcription 3.

The role of airway-resident microbiota in the development of PAH should not be ignored when a growing number of studies are focusing on the effect of gut microbiota on PAH. The proportion of *Streptococcus* in the gut microbiota has been reported to be elevated in both PAH patients and animal models (Kim et al., 2020; Sharma et al., 2020b). Similarly, *Streptococcus* in the airway microbiota was significantly associated with PAH (Zhang et al., 2020) (Table 1). Tsay et al. found that supraglottic dominant taxa, such as *Streptococcus, Veillonella*, and *Prevotella*, were enriched in the lower airways of lung cancer patients and were associated with the upregulation of ERK and phosphatidylinositol 3-kinase (PI3K) signaling pathways in lung cancer (Tsay et al., 2018). Various stimuli such as platelet-derived growth factor (PDGF) induce proliferation of PASMCs and pulmonary vascular remodeling through the PI3K/protein kinase B (AKT)/mammalian target of rapamycin (mTOR) signaling pathway (Ogawa et al., 2012). A clinical trial on airway microbiota remodeling in transplanted lungs found that *Streptococcus* and *Prevotella* were associated with elevated expression of thrombospondin 1 (THBS1) (Mouraux et al., 2018). TSP-1 (THBS1-encoded protein) promotes hypoxia-induced pulmonary vasoconstriction through activation of transforming growth factor-β (TGF-β) signaling (Kumar et al., 2020). Moreover, enrichment of *Veillonella* and *Prevotella* in the pulmonary microbiota was positively correlated with high levels of several cytokines, such as IL-1α, IL-1β, IL-6, fractalkine, and IL-17 (Segal et al., 2016). Interestingly, *Prevotella* has been reported to be reduced in the upper airway microbiota of patients with PAH, asthma, and chronic obstructive pulmonary disease, which may be related to differences in the distribution of the microbiota in different parts of the airway (Hilty et al., 2010; Zhang et al., 2020). It has also been demonstrated that commensal *Prevotella* can induce neutrophilia, chemokine, and cytokine production, but this limited inflammation is well tolerated in murine airways and provokes no detectable lung pathology (Larsen et al., 2015). Therefore, further studies are needed to determine the promotional or protective effects of *Prevotella* on lung diseases such as PAH. *Streptococcus* has also been reported to activate the p38 mitogen-activated protein kinase (MAPK) signaling pathway in primary human bronchial epithelial cells, inducing an increase in IL-6 production (Goleva et al., 2017). And IL-6 may be involved in the proliferation of PASMCs and fibroblasts through the transcription 3 (STAT3) pathway, p38MAPK inhibitor reversed hypoxia and MCT-induced PAH (Church et al., 2015). In addition, *Streptococcus* can also produce butyrate to promote 5-HT production (Butler et al., 2019; Chang et al., 2020) (Figure 2). Zhang et al. found elevated proportions of *Leptotrichiaceae, Lautropia*, and *Ralstonia* in the airways of patients with PAH compared to reference subjects, while less research has been conducted regarding their role (Zhang et al., 2020) (Table 1). Available studies suggest that *Leptotrichia* is associated with arterial stiffness in the elderly (Lee et al., 2019).

## Prospects of Microbiota in the Treatment of PAH

In summary, dysbiosis of the gut and airway is closely related to the pathogenesis of PAH. Thus, altering the gut and airway microbiota, metabolites, and association with PAH, making the microbiota a new therapeutic target for PAH. Therapeutic interventions to eliminate pathogenic microorganisms with antibiotics are commonly used in clinical practice. Antibiotic therapy, a cocktail-containing ampicillin, vancomycin, neomycin, and metronidazole, was performed to treat SU5416/hypoxic rats, and was reported to induce alterations in the gut microbiota with increased abundance of *Proteobacteria* at the phylum level and *Shigella* at genus level (Sanada et al., 2020). Consequently, antibiotic cocktail therapy inhibited the SU5416/hypoxia-induced progression of pulmonary vascular remodeling and the development of PAH in rats. However, there is a consensus that such non-specific antimicrobial therapies may produce many side effects, which may destroy the diversity of intestinal and airway microbiota, leading to multiple infections and drug resistance of harmful microorganisms (Forslund et al., 2013; Rashid et al., 2015).

Probiotics are commonly used to balance the intestinal flora and are currently defined by the Food and Agriculture Organization of the United Nations and the World Health Organization as “live microorganisms in sufficient numbers to provide health benefits to the host” (Hill et al., 2014). A clinical trial showed that supplementation with *Lactobacillus plantarum 299v* reduced vascular endothelial disorders and systemic inflammation in male patients with stable angina by altering the levels of SFCAs (Malik et al., 2018). Wedgwood et al. reported that probiotic treatment with *Lactobacillus reuteri DSM 17938* was able to reverse postnatal growth restriction-induced PAH (Wedgwood et al., 2020). In addition, some researchers reported that the therapeutic scope of probiotics has been expanded by genetic engineering, called engineered probiotics. Currently, engineered probiotics are more researched and effective in metabolic and infectious diseases (Zhou et al., 2020). However, the potential of engineered probiotics in the treatment of PAH needs to be further explored. Although most of the commensal bacteria used as probiotics are from the gastrointestinal tract, commensal bacteria from the respiratory system have also been reported as probiotics. It has been shown that the pro-Th1 strain *CNCM I 4969* (patented strains) isolated from the lungs of neonatal mice can modulate susceptibility to asthma in mice by intranasal inoculation (Remot et al., 2017).

Another strategy for regulating the gut microbiota is prebiotic therapy, which is a substrate selectively utilized by host microbes for the benefit of host health (Gibson et al., 2017). Feng et al. found that algal oligosaccharides could exert anti-inflammatory and antioxidant effects as prebiotics and improve MCT-induced PAH in rats (Feng et al., 2020). Furthermore, algal polysaccharides such as fucoidan, laminarin, alginate, ulvan, and porphyran can increase the production of SFCAs (Feng et al., 2020; Shannon et al., 2021). The International Scientific Association for Probiotics and Prebiotics defines polyunsaturated fatty acids (PUFA) as candidate prebiotics (Gibson et al., 2017). It also has been reported that lipid emulsion containing *n* – 3 PUFA may induce a potent and sustained vasodilatation in the fetal lung (Houeijeh et al., 2011). Furthermore, maternal omega-3 PUFA supplementation prevents hyperoxia-induced PAH in the offspring by suppressing proinflammatory cytokines production, angiogenesis, and vascular remodeling (Zhong et al., 2018). Oral administration of docosapentaenoic acid monoacylglyceride, the re-esterified form of docosapentaenoic acid with monoglycerides with better bioavailability, suppresses inflammation and vascular remodeling and prevents the progression of MCT-induced PAH (Morin et al., 2014; Sztuka et al., 2018). Thus, the intake of probiotics and prebiotics or a combination of both, i.e., synbiotics, may help prevent and treat PAH. However, due to the large individual differences in microbial composition in humans, standardization of the dose, and composition of prebiotic products has become a major challenge that needs to be addressed in larger studies (Suez et al., 2019).

Fecal microbial transplantation (FMT) is a possible therapeutic intervention to regulate the gut microbiota, designed to introduce the fecal contents of a healthy donor into the patient's gastrointestinal tract. This treatment has been evolving, with more research especially in the treatment of intestinal diseases. A landmark clinical trial has shown that FMT has a significant advantage over antibiotics in the treatment of recurrent *Clostridium difficile* infection (van Nood et al., 2013). In recent years, researchers have also begun to pay attention to the utility of FMT for treating diseases related to gut microbial dysbiosis, such as obesity, hypertension, and diabetes (Leshem et al., 2019). Wang et al. reported that FMT reduced intestinal permeability and improved systemic inflammation in rats with hepatic encephalopathy (Wang et al., 2017). As is reported, angiotensin converting enzyme 2 (ACE2) overexpression or FMT from ACE2 overexpressing mice rebalanced the gut microbiota and improved hypoxia-induced PAH, suggesting that ACE2 may improve PAH by regulating the gut microbiota in addition to mediating the renin-angiotensin system (Sharma et al., 2020a). Consequently, we speculate that FMT can also be used for the treatment of PAH, but its efficacy needs to be clarified by a large number of preclinical studies and clinical trials.

In addition, a growing number of studies suggest that mesenchymal stem cells (MSCs) therapy may also improve PAH. A meta-analysis has been conducted to evaluate the therapeutic efficacy of MSCs and secretome in PAH, and concluded that both secretome and MSCs significantly improved PAH by reducing right ventricular systolic pressure, mean pulmonary arterial pressure, and right ventricular remodeling (Muhammad et al., 2020). It has been proved that MSCs therapy could increase capillary density, suppress cardiomyocytes hypertrophy and fibrosis, and improve immunomodulatory activities in PAH (Muhammad et al., 2020). Moreover, it is also reported that MSCs may also improve hypoxia-induced PAH by balancing the intestinal microbiota in mice, and *Micrococcaales, Nesterenkonia, Anaerotruncus*, and *Tyzzerella* may be discriminative and serve as gut microbiota biomarkers of MSCs-treated mice (Luo et al., 2021).

## Conclusions and Future Perspectives

Observational clinical studies and basic research point out that there is a strong association between dysbiosis in the gut and airway and PAH. Reduced microbial diversity has been observed in the gut and airway of PAH. Imbalances between beneficial bacteria such as *Bacteroids* and SCFAs-producing bacteria, and potential pathogenic bacteria such as TMA/TMAO-associated bacteria, are in parallel with PAH, with functional changes of microbiome. These population-based studies of microbiota in PAH are cross-sectional observational studies; and the causal relationship between dysbiosis and PAH could not be established. However, based on animal experiments, we could draw a conclusion that PAH indeed affects the gut microbiome, because the different profiles of gut microbiota were found after exposure to factors such as hypoxia and MCT, and PAH models were established successfully. Interestingly, to explore whether discriminative microbiota has a causal role in PAH, fecal matter transfer from ACE2 knock-in mice, which exhibits distinct gut microbial communities and are protected from developing PAH, ameliorates hypoxia-induced cardiopulmonary hemodynamics and pulmonary vascular remodeling (Sharma et al., 2020a). Taking together, we thought that there is an interactive relationship between altered microbial communities and PAH.

The altered gut/airway microbiota has vital effects on the progress of PAH primarily through modulation of immunity, SCFAs production, polysaccharide fermentation, gut barrier fortification, exacerbation lung infections, and lung/intestine inflammation. However, the causal relationship between the microbiome and the development of PAH is not yet clear and needs to be validated by additional animal studies, prospective cohort studies, and *in vitro* microbiome dysregulation and analysis. Since the living environment and genetic factors can influence the composition of the microbiota *in vivo*, special attention should be paid to improving *in vivo* animal models to simulate human clinical conditions as much as possible. Existing studies provide evidence for entero-pulmonary connection in PAH and suggest that there is overlap between intestinal and pulmonary microbiota, indicating that they may coordinate or antagonize each other in the development of PAH. However, only one research reported the effect of oropharyngeal microbiota on PAH. Although there is a great similarity between the microbiota of the lung and the upper respiratory tract, differences in the distribution of microbiota in different parts of the respiratory tract should be taken into account when studying the lung microbiota, and it may be more important to study the effect of the lung microbiota on PAH. Therefore, more researches are needed to reveal the link between the pulmonary microbiota and the gut microbiota and their role in the pathogenesis of PAH.

Microbiota-based therapy may become a new therapeutic strategy for PAH, but there is a lack of relevant clinical evidence. Large-scale clinical studies need to be designed to validate the effectiveness of microbiota-based therapies for PAH. For example, whether patients who undergo colectomy are at risk for altered PAH and whether patients with PAH treated with FMT or probiotics have improved signs and symptoms of PAH. Besides focusing on bacteria, we should also pay attention to the effect of other microorganisms such as archaea, fungi, and viruses on PAH. It has been shown that bacteria in the gut have strong interactions with phages (Kim S. et al., 2020). Ackermann et al. found severe endothelial damage and thrombosis associated with intracellular severe acute respiratory syndrome coronavirus 2 in coronavirus disease 2019, which is related to the pathogenesis of certain types of PAH (Ackermann et al., 2020).

## Author Contributions

LLH, HDZ, YJL, and YL were involved in conceptualizing and drafting the manuscript. LLH and HDZ were involved in preparing the original draft. HL and YJL revised the manuscript. HDZ and YJL were involved in creating the images and tables. YL reviewed the manuscript. All authors have read and agreed to the published version of the manuscript.

## Funding

This work was supported by the National Natural Science Foundation of China (No. 82171860) and the Luzhou-Southwest Medical University cooperation project (No. 2019LZXNYDJ35).

## Conflict of Interest

The authors declare that the research was conducted in the absence of any commercial or financial relationships that could be construed as a potential conflict of interest.

## Publisher's Note

All claims expressed in this article are solely those of the authors and do not necessarily represent those of their affiliated organizations, or those of the publisher, the editors and the reviewers. Any product that may be evaluated in this article, or claim that may be made by its manufacturer, is not guaranteed or endorsed by the publisher.

## Abbreviations

PAH: pulmonary arterial hypertension
BMPR2: bone morphogenetic protein receptor 2
IL: interleukin
HIF-1α: hypoxia-inducible factor-1α
VEGF: vascular endothelial growth factor
CCR: chemokine receptor
HMGB1: high mobility group box-1
TLR4: toll-like receptor 4
VCAM-1: vascular cell adhesion molecule-1
MCT: monocrotaline
SCFAs: short-chain fatty acids
GPCRs: G protein-coupled receptors
HDAC: histone deacetylase
Tregs: regulatory T cells
LPS: lipopolysaccharide
Th17: T helper 17
5-HT: serotonin
ROS: reactive oxygen species
TMA: trimethylamine
TMAO: trimethylamine N-oxide
PKC: protein kinase C
NF-κB: nuclear factor-kappa B
NLRP3: nod-like receptor family pyrin domain containing 3
SIRT3: sirtuin 3
SOD2: superoxide dismutase 2
ERK1/2: extracellular signal-regulated kinase 1/2
Drp1: dynamin-related protein 1
PI3K: phosphatidylinositol 3-kinase
PDGF: platelet-derived growth factor
AKT: protein kinase B
mTOR: mammalian target of rapamycin
THBS1: thrombospondin 1
TSP-1: THBS1-encoded protein
TGF-β: transforming growth factor-β
MAPK: mitogen-activated protein kinase
STAT3: transcription 3
FAO/WHO: the Food and Agriculture Organization of the United Nations and the WHO
PNGR: postnatal growth restriction
FMT: fecal microbial transplantation
ACE2: angiotensin converting enzyme 2
PUFA: polyunsaturated fatty acids
MSCs: mesenchymal stem cells
SARS-CoV-2: severe acute respiratory syndrome coronavirus 2
COVID-19: coronavirus disease 2019.

## References

[B1] Abid S. Marcos E. Parpaleix A. Amsellem V. Breau M. Houssaini A.. (2019). CCR2/CCR5-mediated macrophage-smooth muscle cell crosstalk in pulmonary hypertension. Eur. Respir. J. 54, 1802308. 10.1183/13993003.02308-2018 31320454

[B2] Ackermann M. Verleden S. E. Kuehnel M. Haverich A. Welte T. Laenger F.. (2020). Pulmonary vascular endothelialitis, thrombosis, and angiogenesis in Covid-19. N. Engl. J. Med. 383, 120–128. 10.1056/NEJMoa2015432 32437596 PMC7412750

[B3] Almeida A. Mitchell A. L. Boland M. Forster S. C. Gloor G. B. Tarkowska A.. (2019). A new genomic blueprint of the human gut microbiota. Nature 568, 499–504. 10.1038/s41586-019-0965-1 30745586 PMC6784870

[B4] Arpaia N. Campbell C. Fan X. Dikiy S. van der Veeken J. deRoos P.. (2013). Metabolites produced by commensal bacteria promote peripheral regulatory T-cell generation. Nature 504, 451–455. 10.1038/nature12726 24226773 PMC3869884

[B5] Bassis C. M. Erb-Downward J. R. Dickson R. P. Freeman C. M. Schmidt T. M. Young V. B.. (2015). Analysis of the upper respiratory tract microbiotas as the source of the lung and gastric microbiotas in healthy individuals. mBio 6, e00037. 10.1128/mBio.00037-15 25736890 PMC4358017

[B6] Beshay S. Sahay S. Humbert M. (2020). Evaluation and management of pulmonary arterial hypertension. Respir. Med. 171, 106099. 10.1016/j.rmed.2020.106099 32829182

[B7] Bowerman K. L. Rehman S. F. Vaughan A. Lachner N. Budden K. F. Kim R. Y.. (2020). Disease-associated gut microbiome and metabolome changes in patients with chronic obstructive pulmonary disease. Nat. Commun. 11, 5886. 10.1038/s41467-020-19701-0 33208745 PMC7676259

[B8] Brittain E. L. Talati M. Fortune N. Agrawal V. Meoli D. F. West J.. (2019). Adverse physiologic effects of Western diet on right ventricular structure and function: role of lipid accumulation and metabolic therapy. Pulm. Circ. 9, 2045894018817741. 10.1177/2045894018817741 30451070 PMC6295706

[B9] Budden K. F. Shukla S. D. Rehman S. F. Bowerman K. L. Keely S. Hugenholtz P.. (2019). Functional effects of the microbiota in chronic respiratory disease. Lancet. Respirat. Med. 7, 907–920. 10.1016/S2213-2600(18)30510-1 30975495

[B10] Butler M. I. Cryan J. F. Dinan T. G. (2019). Man and the microbiome: a new theory of everything? Annu. Rev. Clin. Psychol. 15, 371–398. 10.1146/annurev-clinpsy-050718-095432 30786244

[B11] Callejo M. Mondejar-Parreño G. Barreira B. Izquierdo-Garcia J. L. Morales-Cano D. Esquivel-Ruiz S.. (2018). Pulmonary arterial hypertension affects the rat gut microbiome. Sci. Rep. 8, 9681. 10.1038/s41598-018-27682-w 29946072 PMC6018770

[B12] Chang Y. Chen Y. Zhou Q. Wang C. Chen L. Di W.. (2020). Short-chain fatty acids accompanying changes in the gut microbiome contribute to the development of hypertension in patients with preeclampsia. Clin. Sci. 134, 289–302. 10.1042/CS20191253 31961431

[B13] Chen K. Zheng X. Feng M. Li D. Zhang H. (2017). Gut microbiota-dependent metabolite trimethylamine N-oxide contributes to cardiac dysfunction in western diet-induced obese mice. Front. Physiol. 8, 139. 10.3389/fphys.2017.00139 28377725 PMC5359299

[B14] Chen L. Sun M. Wu W. Yang W. Huang X. Xiao Y.. (2019). Microbiota metabolite butyrate differentially regulates Th1 and Th17 cells' differentiation and function in induction of colitis. Inflamm. Bowel Dis. 25, 1450–1461. 10.1093/ibd/izz046 30918945 PMC6701512

[B15] Chen M. L. Zhu X. H. Ran L. Lang H. D. Yi L. Mi M. T.. (2017). Trimethylamine-N-oxide induces vascular inflammation by activating the NLRP3 inflammasome through the SIRT3-SOD2-mtROS signaling pathway. J. Am. Heart Assoc. 6, e006347. 10.1161/JAHA.117.006347 28871042 PMC5634285

[B16] Church A. C. Martin D. H. Wadsworth R. Bryson G. Fisher A. J. Welsh D. J.. (2015). The reversal of pulmonary vascular remodeling through inhibition of p38 MAPK-alpha: a potential novel anti-inflammatory strategy in pulmonary hypertension. Am. J. Physiol. Lung Cell. Mol. Physiol. 309, L333–L347. 10.1152/ajplung.00038.2015 26024891 PMC4538235

[B17] Cui X. Ye L. Li J. Jin L. Wang W. Li S.. (2018). Metagenomic and metabolomic analyses unveil dysbiosis of gut microbiota in chronic heart failure patients. Sci. Rep. 8, 635. 10.1038/s41598-017-18756-2 29330424 PMC5766622

[B18] Feng W. Hu Y. An N. Feng Z. Liu J. Mou J.. (2020). Alginate oligosaccharide alleviates monocrotaline-induced pulmonary hypertension via anti-oxidant and anti-inflammation pathways in rats. Int. Heart J. 61, 160–168. 10.1536/ihj.19-096 31956132

[B19] Feng W. Wang J. Yan X. Zhang Q. Chai L. Wang Q.. (2021). ERK/Drp1-dependent mitochondrial fission contributes to HMGB1-induced autophagy in pulmonary arterial hypertension. Cell Prolif. 54, e13048. 10.1111/cpr.13048 33948998 PMC8168414

[B20] Forslund K. Sunagawa S. Kultima J. R. Mende D. R. Arumugam M. Typas A.. (2013). Country-specific antibiotic use practices impact the human gut resistome. Genome Res. 23, 1163–1169. 10.1101/gr.155465.113 23568836 PMC3698509

[B21] Gawrys-Kopczynska M. Konop M. Maksymiuk K. Kraszewska K. Derzsi L. Sozanski K.. (2020). TMAO, a seafood-derived molecule, produces diuresis and reduces mortality in heart failure rats. eLife, 9, e57028. 10.7554/eLife.57028.sa2 32510330 PMC7334024

[B22] Gibson G. R. Hutkins R. Sanders M. E. Prescott S. L. Reimer R. A. Salminen S. J.. (2017). Expert consensus document: The International Scientific Association for Probiotics and Prebiotics (ISAPP) consensus statement on the definition and scope of prebiotics. Nat. Rev. Gastroenterol. Hepatol. 14, 491–502. 10.1038/nrgastro.2017.75 28611480

[B23] Goldenberg N. M. Hu Y. Hu X. Volchuk A. Zhao Y. D. Kucherenko M. M.. (2019). Therapeutic targeting of of high-mobility group Box-1 in pulmonary arterial hypertension. Am. J. Respir. Crit. Care Med. 199, 1566–1569. 10.1164/rccm.201808-1597LE 30939030 PMC7125426

[B24] Goleva E. Harris J. K. Robertson C. E. Jackson L. P. Martin R. J. Leung D. Y. M.. (2017). Airway microbiome and responses to corticosteroids in corticosteroid-resistant asthma patients treated with acid suppression medications. J. Allergy Clin. Immunol. 140, 860–862.e1. 10.1016/j.jaci.2017.03.011 28477847

[B25] Gräf S. Haimel M. Bleda M. Hadinnapola C. Southgate L. Li W.. (2018). Identification of rare sequence variation underlying heritable pulmonary arterial hypertension. Nat. Commun. 9, 1416. 10.1038/s41467-018-03672-4 29650961 PMC5897357

[B26] Groves H. T. Cuthbertson L. James P. Moffatt M. F. Cox M. J. Tregoning J. S.. (2018). Respiratory disease following viral lung infection alters the murine gut microbiota. Front. Immunol. 9, 182. 10.3389/fimmu.2018.00182 29483910 PMC5816042

[B27] Guasti L. Galliazzo S. Molaro M. Visconti E. Pennella B. Gaudio G. V.. (2021). TMAO as a biomarker of cardiovascular events: a systematic review and meta-analysis. Intern. Emerg. Med. 16, 201–207. 10.1007/s11739-020-02470-5 32779113

[B28] Halfvarson J. Brislawn C. J. Lamendella R. Vázquez-Baeza Y. Walters W. A. Bramer L. M.. (2017). Dynamics of the human gut microbiome in inflammatory bowel disease. Nat. Microbiol. 2, 17004. 10.1038/nmicrobiol.2017.4 28191884 PMC5319707

[B29] Hayase E. Jenq R. R. (2020). Too much TMAO and GVHD. Blood 136, 383–385. 10.1182/blood.2020006104 32702126 PMC7378460

[B30] He Y. Wen Q. Yao F. Xu D. Huang Y. Wang J.. (2017). Gut-lung axis: the microbial contributions and clinical implications. Crit. Rev. Microbiol. 43, 81–95. 10.1080/1040841X.2016.1176988 27781554

[B31] Hill C. Guarner F. Reid G. Gibson G. R. Merenstein D. J. Pot B.. (2014). Expert consensus document. The International Scientific Association for Probiotics and Prebiotics consensus statement on the scope and appropriate use of the term probiotic. Nat. Rev. Gastroenterol. Hepatol. 11, 506–514. 10.1038/nrgastro.2014.66 24912386

[B32] Hilty M. Burke C. Pedro H. Cardenas P. Bush A. Bossley C.. (2010). Disordered microbial communities in asthmatic airways. PLoS ONE 5, e8578. 10.1371/journal.pone.0008578 20052417 PMC2798952

[B33] Hong W. Mo Q. Wang L. Peng F. Zhou Y. Zou W.. (2021). Changes in the gut microbiome and metabolome in a rat model of pulmonary arterial hypertension. Bioengineered 12, 5173–5183. 10.1080/21655979.2021.1952365 34405758 PMC8806624

[B34] Hood K. Y. Mair K. M. Harvey A. P. Montezano A. C. Touyz R. M. MacLean M. R.. (2017). Serotonin signaling through the 5-HT(1B) receptor and NADPH oxidase 1 in pulmonary arterial hypertension. Arterioscler. Thromb. Vasc. Biol. 37, 1361–1370. 10.1161/ATVBAHA.116.308929 28473438 PMC5478178

[B35] Houeijeh A. Aubry E. Coridon H. Montaigne K. Sfeir R. Deruelle P.. (2011). Effects of n-3 polyunsaturated fatty acids in the fetal pulmonary circulation. Crit. Care Med. 39, 1431–1438. 10.1097/CCM.0b013e31821204fb 21378553

[B36] Jose A. Apewokin S. Hussein W. E. Ollberding N. J. Elwing J. M. Haslam D. B.. (2022). A unique gut microbiota signature in pulmonary arterial hypertension: a pilot study. Pulm. Circ. 12, e12051. 10.1002/pul2.12051 35506110 PMC9052999

[B37] Karoor V. Strassheim D. Sullivan T. Verin A. Umapathy N. S. Dempsey E. C.. (2021). The short-chain fatty acid butyrate attenuates pulmonary vascular remodeling and inflammation in hypoxia-induced pulmonary hypertension. Int. J. Mol. Sci. 22, 9916. 10.3390/ijms22189916 34576081 PMC8467617

[B38] Kemter A. M. Nagler C. R. (2019). Influences on allergic mechanisms through gut, lung, and skin microbiome exposures. J. Clin. Invest. 129, 1483–1492. 10.1172/JCI124610 30830878 PMC6436857

[B39] Kim M. H. Yun K. E. Kim J. Park E. Chang Y. Ryu S.. (2020). Gut microbiota and metabolic health among overweight and obese individuals. Sci. Rep. 10, 19417. 10.1038/s41598-020-76474-8 33173145 PMC7655835

[B40] Kim S. Rigatto K. Gazzana M. B. Knorst M. M. Richards E. M. Pepine C. J.. (2020). Altered gut microbiome profile in patients with pulmonary arterial hypertension. Hypertension 75, 1063–1071. 10.1161/HYPERTENSIONAHA.119.14294 32088998 PMC7067661

[B41] Kim S. H. Kim K. W. Jeong J. W. (2007). Inhibition of hypoxia-induced angiogenesis by sodium butyrate, a histone deacetylase inhibitor, through hypoxia-inducible factor-1alpha suppression. Oncol. Rep. 17, 793–797. 10.3892/or.17.4.793 17342317

[B42] Kim S. S. Walejko J. Rigatto K. Jordan R. Shapiro B. Pepine C.. (2018). Abstract P167: altered gut microbiome contributes to metabolite biomarkers in patients with pulmonary hypertension. Hypertension 72, AP167. 10.1161/hyp.72.suppl_1.P167

[B43] Koliada A. Syzenko G. Moseiko V. Budovska L. Puchkov K. Perederiy V.. (2017). Association between body mass index and Firmicutes/Bacteroidetes ratio in an adult Ukrainian population. BMC Microbiol. 17, 120. 10.1186/s12866-017-1027-1 28532414 PMC5440985

[B44] Kumar R. Mickael C. Kassa B. Sanders L. Hernandez-Saavedra D. Koyanagi D. E.. (2020). Interstitial macrophage-derived thrombospondin-1 contributes to hypoxia-induced pulmonary hypertension. Cardiovasc. Res. 116, 2021–2030. 10.1093/cvr/cvz304 31710666 PMC7519884

[B45] Larsen J. M. Musavian H. S. Butt T. M. Ingvorsen C. Thysen A. H. Brix S.. (2015). Chronic obstructive pulmonary disease and asthma-associated Proteobacteria, but not commensal *Prevotella* spp., promote Toll-like receptor 2-independent lung inflammation and pathology. Immunology 144, 333–342. 10.1111/imm.12376 25179236 PMC4298427

[B46] Lawrie A. Hameed A. G. Chamberlain J. Arnold N. Kennerley A. Hopkinson K.. (2011). Paigen diet-fed apolipoprotein E knockout mice develop severe pulmonary hypertension in an interleukin-1-dependent manner. Am. J. Pathol. 179, 1693–1705. 10.1016/j.ajpath.2011.06.037 21835155 PMC3181351

[B47] Le Hiress M. Tu L. Ricard N. Phan C. Thuillet R. Fadel E.. (2015). Proinflammatory signature of the dysfunctional endothelium in pulmonary hypertension. Role of the macrophage migration inhibitory factor/CD74 complex. Am. J. Respirat. Crit. Care Med. 192, 983–97. 10.1164/rccm.201402-0322OC 26203495

[B48] Leber L. Beaudet A. Muller A. (2021). Epidemiology of pulmonary arterial hypertension and chronic thromboembolic pulmonary hypertension: identification of the most accurate estimates from a systematic literature review. Pulm. Circ. 11, 2045894020977300. 10.1177/2045894020977300 33456755 PMC7797595

[B49] Lee S. Y. Mac Aogáin M. Fam K. D. Chia K. L. Binte Mohamed Ali N. A. Yap M. M. C.. (2019). Airway microbiome composition correlates with lung function and arterial stiffness in an age-dependent manner. PLoS ONE 14, e0225636. 10.1371/journal.pone.0225636 31770392 PMC6879132

[B50] Leshem A. Horesh N. Elinav E. (2019). Fecal microbial transplantation and its potential application in cardiometabolic syndrome. Front. Immunol. 10, 1341. 10.3389/fimmu.2019.01341 31258528 PMC6587678

[B51] Li J. Zhao F. Wang Y. Chen J. Tao J. Tian G.. (2017). Gut microbiota dysbiosis contributes to the development of hypertension. Microbiome 5, 14. 10.1186/s40168-016-0222-x 28143587 PMC5286796

[B52] Li M. van Esch B. Henricks P. A. J. Folkerts G. Garssen J. (2018). The anti-inflammatory effects of short chain fatty acids on lipopolysaccharide- or tumor necrosis factor α-stimulated endothelial cells via activation of GPR41/43 and inhibition of HDACs. Front. Pharmacol. 9, 533. 10.3389/fphar.2018.00533 29875665 PMC5974203

[B53] Liu J. Wang W. Wang L. Chen S. Tian B. Huang K.. (2018). IL-33 Initiates vascular remodelling in hypoxic pulmonary hypertension by up-regulating HIF-1α and VEGF expression in vascular endothelial cells. EBioMedicine 33, 196–210. 10.1016/j.ebiom.2018.06.003 29921553 PMC6085568

[B54] Liu Q. Tian X. Maruyama D. Arjomandi M. Prakash A. (2021). Lung immune tone via gut-lung axis: gut-derived LPS and short-chain fatty acids' immunometabolic regulation of lung IL-1β, FFAR2, and FFAR3 expression. Am. J. Physiol. Lung Cell. Mol. Physiol. 321, L65–l78. 10.1152/ajplung.00421.2020 33851870 PMC8321849

[B55] Long L. MacLean M. R. Jeffery T. K. Morecroft I. Yang X. Rudarakanchana N.. (2006). Serotonin increases susceptibility to pulmonary hypertension in BMPR2-deficient mice. Circ. Res. 98, 818–827. 10.1161/01.RES.0000215809.47923.fd 16497988

[B56] Luo L. Chen Q. Yang L. Zhang Z. Xu J. Gou D.. (2021). MSCs therapy reverse the gut microbiota in hypoxia-induced pulmonary hypertension mice. Front. Physiol. 12, 712139. 10.3389/fphys.2021.712139 34531759 PMC8438532

[B57] Ma G. Pan B. Chen Y. Guo C. Zhao M. Zheng L.. (2017). Trimethylamine N-oxide in atherogenesis: impairing endothelial self-repair capacity and enhancing monocyte adhesion. Biosci. Rep. 37, BSR20160244. 10.1042/BSR20160244 28153917 PMC5333780

[B58] Malik M. Suboc T. M. Tyagi S. Salzman N. Wang J. Ying R.. (2018). *Lactobacillus plantarum* 299v supplementation improves vascular endothelial function and reduces inflammatory biomarkers in men with stable coronary artery disease. Circ. Res. 123, 1091–1102. 10.1161/CIRCRESAHA.118.313565 30355158 PMC6205737

[B59] Mariat D. Firmesse O. Levenez F. Guimarăes V. Sokol H. Doré J.. (2009). The Firmicutes/Bacteroidetes ratio of the human microbiota changes with age. BMC Microbiol. 9, 123. 10.1186/1471-2180-9-123 19508720 PMC2702274

[B60] Morin C. Hiram R. Rousseau E. Blier P. U. Fortin S. (2014). Docosapentaenoic acid monoacylglyceride reduces inflammation and vascular remodeling in experimental pulmonary hypertension. Am. J. Physiol. Heart Circ. Physiol. 307, H574–H586. 10.1152/ajpheart.00814.2013 24929859

[B61] Mouraux S. Bernasconi E. Pattaroni C. Koutsokera A. Aubert J. D. Claustre J.. (2018). Airway microbiota signals anabolic and catabolic remodeling in the transplanted lung. J. Allergy Clin. Immunol. 141, 718–729.e7. 10.1016/j.jaci.2017.06.022 28729000 PMC5792246

[B62] Muhammad S. A. Abbas A. Y. Saidu Y. Fakurazi S. Bilbis L. S. (2020). Therapeutic efficacy of mesenchymal stromal cells and secretome in pulmonary arterial hypertension: a systematic review and meta-analysis. Biochimie 168, 156–168. 10.1016/j.biochi.2019.10.016 31678635

[B63] Ni S. Ji T. Dong J. Chen F. Feng H. Zhao H.. (2022). Immune cells in pulmonary arterial hypertension. Heart Lung Circ. 31, 934–943. 10.1016/j.hlc.2022.02.007 35361533

[B64] Ogawa A. Firth A. L. Smith K. A. Maliakal M. V. Yuan J. X. (2012). PDGF enhances store-operated Ca2+ entry by upregulating STIM1/Orai1 via activation of Akt/mTOR in human pulmonary arterial smooth muscle cells. Am. J. Physiol,. Cell Physiol. 302, C405–C411. 10.1152/ajpcell.00337.2011 22031597 PMC3328839

[B65] Parpaleix A. Amsellem V. Houssaini A. Abid S. Breau M. Marcos E.. (2016). Role of interleukin-1 receptor 1/MyD88 signalling in the development and progression of pulmonary hypertension. Eur. Respir. J. 48, 470–483. 10.1183/13993003.01448-2015 27418552

[B66] Plöger S. Stumpff F. Penner G. B. Schulzke J. D. Gäbel G. Martens H.. (2012). Microbial butyrate and its role for barrier function in the gastrointestinal tract. Ann. N. Y. Acad. Sci. 1258, 52–59. 10.1111/j.1749-6632.2012.06553.x 22731715

[B67] Poch D. Mandel J. (2021). Pulmonary hypertension. Ann. Intern. Med. 174, Itc49–itc64. 10.7326/AITC202104200 33844574

[B68] Ranchoux B. Bigorgne A. Hautefort A. Girerd B. Sitbon O. Montani D.. (2017). Gut-lung connection in pulmonary arterial hypertension. Am. J. Respir. Cell Mol. Biol. 56, 402–405. 10.1165/rcmb.2015-0404LE 28248132

[B69] Rashid M. U. Zaura E. Buijs M. J. Keijser B. J. Crielaard W. Nord C. E.. (2015). Determining the long-term effect of antibiotic administration on the human normal intestinal microbiota using culture and pyrosequencing methods. Clin. Infect. Dis. 60(Suppl. 2), S77–84. 10.1093/cid/civ137 25922405

[B70] Reigstad C. S. Salmonson C. E. Rainey J. F. 3rd Szurszewski J. H. Linden D. R. Sonnenburg J. L.. (2015). Gut microbes promote colonic serotonin production through an effect of short-chain fatty acids on enterochromaffin cells. FASEB J. 29, 1395–1403. 10.1096/fj.14-259598 25550456 PMC4396604

[B71] Remot A. Descamps D. Noordine M. L. Boukadiri A. Mathieu E. Robert V.. (2017). Bacteria isolated from lung modulate asthma susceptibility in mice. ISME J. 11, 1061–1074. 10.1038/ismej.2016.181 28045458 PMC5437918

[B72] Ruopp N. F. Cockrill B. A. (2022). Diagnosis and treatment of pulmonary arterial hypertension: a review. JAMA 327, 1379–1391. 10.1001/jama.2022.4402 35412560

[B73] Sanada T. J. Hosomi K. Shoji H. Park J. Naito A. Ikubo Y.. (2020). Gut microbiota modification suppresses the development of pulmonary arterial hypertension in an SU5416/hypoxia rat model. Pulm. Circ. 10, 2045894020929147. 10.1177/2045894020929147 32922743 PMC7457673

[B74] Savai R. Pullamsetti S. S. Kolbe J. Bieniek E. Voswinckel R. Fink L.. (2012). Immune and inflammatory cell involvement in the pathology of idiopathic pulmonary arterial hypertension. Am. J. Respir. Crit. Care Med. 186, 897–908. 10.1164/rccm.201202-0335OC 22955318

[B75] Savale L. Akagi S. Tu L. Cumont A. Thuillet R. Phan C.. (2021). Serum and pulmonary uric acid in pulmonary arterial hypertension. Eur. Respir. J. 58, 2000332. 10.1183/13993003.00332-2020 33446602

[B76] Segal L. N. Clemente J. C. Tsay J. C. Koralov S. B. Keller B. C. Wu B. G.. (2016). Enrichment of the lung microbiome with oral taxa is associated with lung inflammation of a Th17 phenotype. Nat. Microbiol. 1, 16031. 10.1038/nmicrobiol.2016.31 27572644 PMC5010013

[B77] Shannon E. Conlon M. Hayes M. (2021). Seaweed components as potential modulators of the gut microbiota. Mar. Drugs 19, 358. 10.3390/md19070358 34201794 PMC8303941

[B78] Sharma R. K. Oliveira A. C. Yang T. Karas M. M. Li J. Lobaton G. O.. (2020a). Gut pathology and its rescue by ACE2 (angiotensin-converting enzyme 2) in hypoxia-induced pulmonary hypertension. Hypertension 76, 206–216. 10.1161/HYPERTENSIONAHA.120.14931 32418496 PMC7505091

[B79] Sharma R. K. Oliveira A. C. Yang T. Kim S. Zubcevic J. Aquino V.. (2020b). Pulmonary arterial hypertension-associated changes in gut pathology and microbiota. ERJ Open Res. 6, 00253-2019. 10.1183/23120541.00253-2019 32743008 PMC7383054

[B80] Shrestha A. K. Menon R. T. El-Saie A. Barrios R. Reynolds C. Shivanna B.. (2020). Interactive and independent effects of early lipopolysaccharide and hyperoxia exposure on developing murine lungs. Am. J. Physiol. Lung Cell. Mol. Physiol. 319, L981–L996. 10.1152/ajplung.00013.2020 32901520 PMC7792686

[B81] Singh G. B. Zhang Y. Boini K. M. Koka S. (2019). High mobility group box 1 mediates TMAO-induced endothelial dysfunction. Int. J. Mol. Sci. 20, 3570. 10.3390/ijms20143570 31336567 PMC6678463

[B82] Smith P. M. Howitt M. R. Panikov N. Michaud M. Gallini C. A. Bohlooly Y. M.. (2013). The microbial metabolites, short-chain fatty acids, regulate colonic Treg cell homeostasis. Science 341, 569–573. 10.1126/science.1241165 23828891 PMC3807819

[B83] Sockrider M. (2021). What is pulmonary hypertension? Am. J. Respir. Crit. Care Med. 203, 12–13. 10.1164/rccm.2035P12 33646084

[B84] Soon E. Crosby A. Southwood M. Yang P. Tajsic T. Toshner M.. (2015). Bone morphogenetic protein receptor type II deficiency and increased inflammatory cytokine production. A gateway to pulmonary arterial hypertension. Am. J. Respirat. Crit. Care Med. 192, 859–872. 10.1164/rccm.201408-1509OC 26073741 PMC4613895

[B85] Stappenbeck T. S. Virgin H. W. (2016). Accounting for reciprocal host-microbiome interactions in experimental science. Nature 534, 191–199. 10.1038/nature18285 27279212

[B86] Suez J. Zmora N. Segal E. Elinav E. (2019). The pros, cons, and many unknowns of probiotics. Nat. Med. 25, 716–729. 10.1038/s41591-019-0439-x 31061539

[B87] Sztormowska-Achranowicz K. Jankowski Z. Kocić I. (2020). Protective effect of nicotinamide and L-arginine against monocrotaline-induced pulmonary hypertension in rats: gender dependence. Pharmacol. Rep.: PR 72, 1334–1346. 10.1007/s43440-020-00125-y 32632916 PMC7550290

[B88] Sztuka K. Orszulak-Michalak D. Jasińska-Stroschein M. (2018). Systematic review and meta-analysis of interventions tested in animal models of pulmonary hypertension. Vascul. Pharmacol. 110, 55–63. 10.1016/j.vph.2018.08.004 30145225

[B89] Tamosiuniene R. Tian W. Dhillon G. Wang L. Sung Y. K. Gera L.. (2011). cells limit vascular endothelial injury and prevent pulmonary hypertension. Circ. Res. 109, 867–879. 10.1161/CIRCRESAHA.110.236927 21868697 PMC3204361

[B90] Tamura Y. Phan C. Tu L. Le Hiress M. Thuillet R. Jutant E. M.. (2018). Ectopic upregulation of membrane-bound IL6R drives vascular remodeling in pulmonary arterial hypertension. J. Clin. Invest. 128, 1956–1970. 10.1172/JCI96462 29629897 PMC5919830

[B91] Thenappan T. Ormiston M. L. Ryan J. J. Archer S. L. (2018). Pulmonary arterial hypertension: pathogenesis and clinical management. BMJ (Clin. Res. Ed.) 360, j5492. 10.1136/bmj.j5492 29540357 PMC6889979

[B92] Trompette A. Gollwitzer E. S. Yadava K. Sichelstiel A. K. Sprenger N. Ngom-Bru C.. (2014). Gut microbiota metabolism of dietary fiber influences allergic airway disease and hematopoiesis. Nat. Med. 20, 159–166. 10.1038/nm.3444 24390308

[B93] Tsai H. J. Tsai W. C. Hung W. C. Hung W. W. Chang C. C. Dai C. Y.. (2021). Gut microbiota and subclinical cardiovascular disease in patients with type 2 diabetes mellitus. Nutrients 13, 2679. 10.3390/nu13082679 34444839 PMC8397936

[B94] Tsay J. J. Wu B. G. Badri M. H. Clemente J. C. Shen N. Meyn P.. (2018). Airway microbiota is associated with upregulation of the PI3K pathway in lung cancer. Am. J. Respir. Crit. Care Med. 198, 1188–1198. 10.1164/rccm.201710-2118OC 29864375 PMC6221574

[B95] van Nood E. Vrieze A. Nieuwdorp M. Fuentes S. Zoetendal E. G. Vos d. e.. (2013). Duodenal infusion of donor feces for recurrent *Clostridium difficile*. N. Engl. J. Med. 368, 407–415. 10.1056/NEJMoa1205037 23323867

[B96] Wang R. R. Yuan T. Y. Wang J. M. Chen Y. C. Zhao J. L. Li M. T.. (2022). Immunity and inflammation in pulmonary arterial hypertension: from pathophysiology mechanisms to treatment perspective. Pharmacol. Res. 180, 106238. 10.1016/j.phrs.2022.106238 35504356

[B97] Wang W. W. Zhang Y. Huang X. B. You N. Zheng L. Li J.. (2017). Fecal microbiota transplantation prevents hepatic encephalopathy in rats with carbon tetrachloride-induced acute hepatic dysfunction. World J. Gastroenterol. 23, 6983–6994. 10.3748/wjg.v23.i38.6983 29097871 PMC5658316

[B98] Wedgwood S. Warford C. Agvatisiri S. R. Thai P. N. Chiamvimonvat N. Kalanetra K. M.. (2020). The developing gut-lung axis: postnatal growth restriction, intestinal dysbiosis, and pulmonary hypertension in a rodent model. Pediatr. Res. 87, 472–479. 10.1038/s41390-019-0578-2 31537010 PMC7035999

[B99] Zanoni I. Ostuni R. Marek L. R. Barresi S. Barbalat R. Barton G. M.. (2011). CD14 controls the LPS-induced endocytosis of Toll-like receptor 4. Cell 147, 868–880. 10.1016/j.cell.2011.09.051 22078883 PMC3217211

[B100] Zhang C. Zhang T. Lu W. Duan X. Luo X. Liu S.. (2020). Altered airway microbiota composition in patients with pulmonary hypertension. Hypertension 76, 1589–1599. 10.1161/HYPERTENSIONAHA.120.15025 32921193

[B101] Zhong Y. Catheline D. Houeijeh A. Sharma D. Du L. Besengez C.. (2018). Maternal omega-3 PUFA supplementation prevents hyperoxia-induced pulmonary hypertension in the offspring. Am. J. Physiol. Lung Cell. Mol. Physiol. 315, L116–L132. 10.1152/ajplung.00527.2017 29597832

[B102] Zhou Z. Chen X. Sheng H. Shen X. Sun X. Yan Y.. (2020). Engineering probiotics as living diagnostics and therapeutics for improving human health. Microb. Cell Fact. 19, 56. 10.1186/s12934-020-01318-z 32131831 PMC7055047

[B103] Zhu F. Zuo L. Hu R. Wang J. Yang Z. Qi X.. (2021). Effect of immune cell infiltration on occurrence of pulmonary hypertension in pulmonary fibrosis patients based on gene expression profiles. Front. Med. 8, 671617. 10.3389/fmed.2021.671617 34307406 PMC8292720

[B104] Zhu W. Gregory J. C. Org E. Buffa J. A. Gupta N. Wang Z.. (2016). AO enhances platelet hyperreactivity and thrombosis risk. Cell 165, 111–124. 10.1016/j.cell.2016.02.011 26972052 PMC4862743

